# Liquid Biopsy and Automated Next-Generation Sequencing: Achieving Results in 27 Hours Within a Community Setting

**DOI:** 10.3390/diagnostics16010037

**Published:** 2025-12-22

**Authors:** Tomomi Yajima, Fumitake Hata, Sei Kurokawa, Kanan Sawamoto, Akiko Yajima, Daisuke Furuya, Noriyuki Sato

**Affiliations:** 1 Department of Surgery, Sapporo Dohto Hospital, 14-3-2 Kita 17-jo Higashi, Higashi-ku, Sapporo 065-0017, Japan; fhata@sdhmc.jp; 2 Department of Clinical Laboratory, Sapporo Dohto Hospital, 14-3-2 Kita 17-jo Higashi, Higashi-ku, Sapporo 065-0017, Japan; k-sawamoto@sdhmc.jp; 3 Department of Internal Medicine, Sapporo Dohto Hospital, 14-3-2 Kita 17-jo Higashi, Higashi-ku, Sapporo 065-0017, Japan; s-kurokawa@sdhmc.jp; 4 Sapporo Female Clinic, 3-2-5 Kita 21-jo Higashi, Higashi-ku, Sapporo 065-0021, Japan; akiko@sapporo-fc.jp; 5 Department of Clinical Engineering, Faculty of Health Sciences, Hokkaido University of Science, 15-4-1 Maeda 7-jo, Teine-ku, Sapporo 006-8585, Japan; furuya-d@hus.ac.jp; 6 Academic Center, Sapporo Dohto Hospital, 14-3-2 Kita 17-jo Higashi, Higashi-ku, Sapporo 065-0017, Japan; nsatou@sapmed.ac.jp

**Keywords:** liquid biopsy, next-generation sequencing, community setting

## Abstract

**Background/Objectives**: Conventional next-generation sequencing (NGS) workflows often require more than two weeks to complete, delaying treatment decisions and limiting access to precision oncology in community settings. This report aimed to demonstrate the feasibility of performing rapid, comprehensive cell-free DNA (cfDNA)-based genomic profiling by introducing a fully automated NGS workflow in a community hospital environment. **Case Presentation**: A postoperative patient with pancreatic ductal adenocarcinoma and liver metastasis underwent cfDNA-based liquid biopsy using plasma collected in PAXgene^®^ Blood ccfDNA Tubes. Gene analysis was performed using the Oncomine Precision Assay GX5 on the Ion Torrent Genexus™ System (Thermo Fisher Scientific). Three pathogenic hotspot mutations—KRAS G12R, TP53 M246I/M246K, and GNA11—and one copy number gain in PIK3CA were identified, whereas no variants were detected in a healthy volunteer control. The total turnaround time from plasma separation to report generation was approximately 27 h, requiring only 40 min of total hands-on time. **Discussion**: This rapid, automated workflow enabled comprehensive cfDNA analysis within a clinically practical timeframe, overcoming key limitations of conventional multi-step NGS workflows that typically require external sample shipment and specialized personnel. The results confirm the technical feasibility of conducting high-quality molecular testing in a regional hospital setting. **Conclusions**: This report demonstrates that fully automated cfDNA-based NGS can achieve clinically meaningful genomic profiling within 27 h in a community hospital. This advancement addresses the time and cost barriers of traditional NGS analysis and represents a significant step toward promoting precision medicine in community healthcare.

## 1. Introduction

Biomarker analysis plays a central role in precision cancer treatment strategies. Next-generation sequencing (NGS) enables rapid, large-scale gene panel analysis, allowing the identification and detection of a wide variety of actionable biomarkers that guide targeted therapy, immunotherapy selection, and prognostic assessment [1,2,3]. In parallel, liquid biopsy using circulating tumor DNA (ctDNA) has gained attention as a noninvasive and repeatable method for genomic testing [4,5]. Comprehensive genomic analysis of ctDNA holds great promise for personalized oncology, with potential applications in targeted drug discovery, early detection of post-operative recurrence and metastasis, tumor burden assessment, minimal residual disease (MRD) evaluation, and early detection of drug resistance [6,7,8,9].

Several institutions have also reported successful implementation of rapid, community-based NGS workflows using automated systems, improving turnaround time and accessibility to molecular testing [10].

Recent studies have further expanded the scope of liquid biopsy beyond plasma analysis, including its use in cerebrospinal fluid, bile, and other body fluids, demonstrating its versatility across various tumor types and disease stages. The Oncomine™ Pan-Cancer Cell-Free Assay, for instance, has been successfully applied to central nervous system malignancies using cerebrospinal fluid samples, detecting ctDNA even when cytology results were negative [11], as well as to bile-derived cfDNA for the early identification of malignant biliary strictures [12]. Moreover, cell-free DNA (cfDNA) profiling has been utilized to assess therapeutic response and resistance mechanisms, such as in EGFR-mutant lung cancer with RBM10 deficiency [13]. These studies underscore the expanding potential of liquid biopsy for minimally invasive genomic profiling and real-time disease monitoring.

Despite its clinical potential, several barriers hinder the widespread adoption of NGS-based biomarker testing. Traditional NGS workflows involve multiple manual processes, such as nucleic acid extraction, library preparation, sequencing, and bioinformatics analysis, often requiring coordination among specialized laboratories. Because of this complexity, the turnaround time for conventional NGS assays remains long. According to the European Society for Medical Oncology (ESMO) Precision Medicine Working Group, most clinical laboratories require more than two weeks to deliver comprehensive genomic reports [14], delaying treatment decisions and limiting the feasibility of repeated monitoring. In addition, manual handling introduces variability in sample quality, turnaround time (TAT), and data reproducibility. The dependence on specialized personnel and centralized testing facilities contributes to regional disparities and increased healthcare costs, posing a significant obstacle to the realization of precision medicine in community-based settings.

In recent years, automation has emerged as a promising solution to overcome these logistical and technical challenges. Fully automated NGS platforms, such as the Ion Torrent Genexus System, composed of a purification module and an integrated sequencer, can streamline cfDNA extraction, library preparation, sequencing, and data analysis, significantly shortening TAT and minimizing operator-dependent variability. Several recent reports have demonstrated that automated systems can produce high-quality sequencing data comparable to those of manual workflows, with total turnaround times within 24 h and hands-on time typically less than one hour [2,5]. In addition, real-world implementation studies have confirmed their feasibility and reliability in community-based molecular testing environments [10]. By simplifying complex laboratory procedures, automation has the potential to decentralize molecular testing and extend genomic profiling capabilities beyond tertiary cancer centers.

Furthermore, the application of liquid biopsy in community hospitals is particularly meaningful for cancers with high recurrence risk, such as pancreatic cancer, where timely detection of molecular relapse can directly influence therapeutic decision-making. Implementing automated NGS within a local hospital infrastructure may enable rapid, in-house reporting of clinically actionable genomic information, facilitating faster transitions between diagnosis, treatment initiation, and relapse monitoring. However, real-world evidence demonstrating the feasibility of such rapid, in-house genomic workflows in non-academic hospital environments remains limited.

We aimed to address these gaps by introducing a fully automated NGS system into a community hospital setting and evaluating its feasibility for liquid biopsy-based biomarker analysis. The goal was to evaluate the feasibility of implementing a fully automated cfDNA-based NGS workflow in a community hospital setting and to describe the unexpectedly rapid TAT achieved in this case report.

## 2. Case Presentation

**Figure 1:**
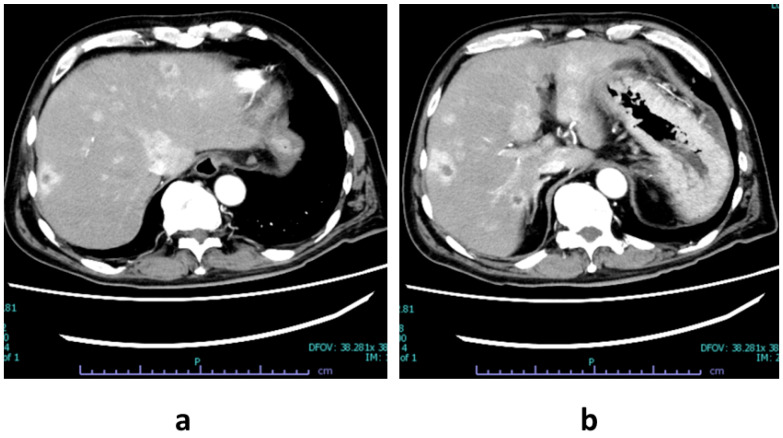
Contrast-enhanced abdominal CT (arterial phase) showing multiple hypovascular liver metastases from recurrent pancreatic ductal adenocarcinoma. (**a**) Axial image demonstrating multiple low-attenuation nodules with peripheral rim enhancement in both hepatic lobes. (**b**) Another slice showing a dominant hypovascular lesion in the right hepatic lobe, also exhibiting a ring-like enhancement pattern, consistent with metastatic disease.

A 67-year-old man with borderline-resectable pancreatic adenosquamous carcinoma underwent neoadjuvant chemotherapy followed by curative resection. The patient had a medical history of benign gastrointestinal lesions that had been treated endoscopically and locally excised cutaneous lesions on the fingers diagnosed as Bowen’s disease during the preceding years. He was not taking any regular medication and had no other chronic illnesses. He had no significant family or genetic history related to cancer. The final pathological diagnosis was stage IB according to the *TNM Classification of Malignant Tumors*, 8th edition, by the Union for International Cancer Control [15]. Three months after surgery, follow-up contrast-enhanced abdominal CT revealed multiple hepatic lesions consistent with metastatic pancreatic ductal adenocarcinoma. These lesions appeared as hypovascular nodules with distinct peripheral rim enhancement during the arterial phase, predominantly located in both hepatic lobes (Figure 1a,b). No local recurrence was identified at the surgical margin, and other organs showed no evidence of metastasis. Systemic chemotherapy with nal-IRI plus 5-FU/LV was scheduled for recurrent disease.

Before initiating chemotherapy, a peripheral blood sample was collected for liquid biopsy analysis to perform rapid cfDNA-based genomic profiling. The patient’s initial diagnosis, preoperative therapy, and curative resection had been performed at a university hospital in Sapporo, Japan. Liquid biopsy analysis for disease recurrence, including cfDNA extraction and NGS, was performed at our community hospital to evaluate the feasibility of implementing automated molecular diagnostics outside a tertiary care setting.

For comparison, one healthy male volunteer in his 50s who had no evidence of malignancy on a recent comprehensive health checkup at our hospital was enrolled as a control. Both participants provided written informed consent prior to sample collection.

Blood samples were collected using PAXgene ccfDNA collection tubes (Becton Dickinson and Company, Tokyo, Japan), which have been shown to stabilize cfDNA and RNA for several days under ambient conditions [16]. Plasma was isolated through a two-step centrifugation protocol using a swing-rotor centrifuge: an initial spin at 3000× *g* for 15 min at room temperature (15–25 °C), followed by transfer of the plasma layer without disturbing the buffy coat, and a second spin at 3000× *g* for 10 min. The clarified plasma was then subjected to fully automated cfDNA extraction on the Ion Torrent Genexus Purification System (Thermo Fisher Scientific, Waltham, MA, USA). Because cfDNA yield from plasma is typically low, the entire eluate was used for library preparation without pre-quantification. Effective cfDNA input was assessed post-sequencing based on molecular coverage metrics generated by the Ion Torrent Genexus Integrated Sequencer (Thermo Fisher Scientific, Waltham, MA, USA); in this case, the median molecular coverage was 3669, which corresponds to an estimated cfDNA mass of approximately a few tens of nanograms, indicating adequate input for variant detection in liquid biopsy applications.

Comprehensive gene analysis was conducted using the Oncomine Precision Assay GX5 (Thermo Fisher Scientific, Waltham, MA, USA) on the Ion Torrent Genexus System (Thermo Fisher Scientific, Waltham, MA, USA), which integrates nucleic acid extraction, library preparation, sequencing, and data analysis in a single automated workflow. Variant annotation and reporting were performed via the Ion Torrent™ Cancer Genomic Analysis Report Service, which utilizes the Oncomine Reporter™ software (version 6.0.2; Thermo Fisher Scientific, Waltham, MA, USA), with annotation data version 2024.01 (006). Variant call files generated by the Ion Torrent Genexus software (version 6.8.1.1; Thermo Fisher Scientific, Waltham, MA, USA) were downloaded and sent to a Thermo Fisher Scientific specialist, who processed the data and generated a structured PDF report summarizing clinical evidence. The findings were jointly reviewed with the specialist to finalize the annotated output.

The start and end times for each process step were automatically recorded in the Genexus software logs. TAT was defined as the interval from blood collection to completion of the genomic report.

In the pancreatic cancer case, three hotspot mutations—*KRAS* G12R, *TP53* M246I/M246K, and *GNA11*—and one copy number gain of *PIK3CA* were detected. No pathogenic alterations were found in the healthy control sample processed under identical conditions.

**Figure 2:**
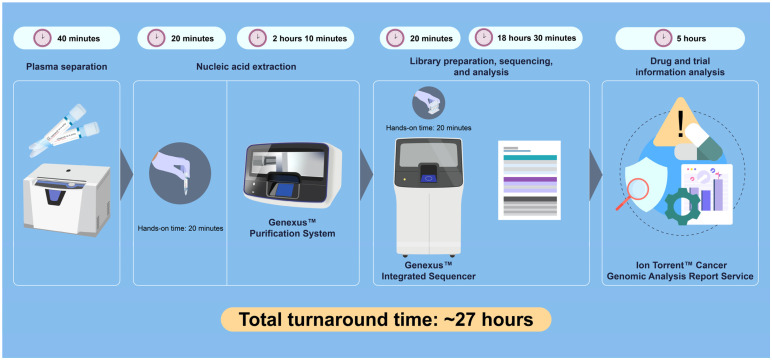
Automated next-generation sequencing workflow and cumulative duration of each step. The figure illustrates the automated next-generation sequencing workflow implemented in a community hospital, showing a total turnaround time of approximately 27 h from plasma separation to obtaining the report. Each step indicates the duration and hands-on time: plasma separation (40 min), nucleic acid extraction (2 h 10 min; hands-on 20 min) using the Genexus™ Purification System, library preparation, sequencing, and analysis (18 h 30 min; hands-on 20 min) using the Genexus™ Integrated Sequencer, and drug and trial information analysis (5 h) via the Ion Torrent™ Cancer Genomic Analysis Report Service.

The automated NGS workflow demonstrated remarkable analytical efficiency: cfDNA extraction required approximately 2 h 10 min, library preparation and sequencing took 18 h 30 min, and data analysis and reporting were completed within 5 h, resulting in a total TAT of approximately 27 h. Hands-on time was limited to about 40 min, primarily for loading samples and reagents. Detected variants and their corresponding allele frequencies are summarized in Table 1, and the overall 27 h workflow is illustrated in Figure 2.

This streamlined, end-to-end process enabled comprehensive genomic profiling to be completed in approximately 27 h within a community hospital laboratory, addressing one of the major limitations of conventional NGS workflows, which typically require more than two weeks to deliver results. These findings highlight the potential for decentralized precision oncology testing without reliance on external sequencing facilities.

A structured therapeutic summary was generated via the Oncomine™ Reporter by submitting the Genexus sequencing results to the Ion Torrent™ Cancer Genomic Analysis Report Service, which integrates global regulatory and guideline databases (FDA, NCCN, EMA, and ESMO) as well as active clinical trial registries based on the detected genomic alterations (Table 2).

## 3. Discussion

To the best of our knowledge, this study is the first to demonstrate that a fully automated NGS system can be successfully implemented in a community hospital setting, enabling comprehensive cfDNA-based genomic profiling to be completed in less than 27 h. This achievement addresses many challenges associated with conventional NGS workflows, which typically require extensive manual handling, specialized expertise, and interfacility specimen transfers, often resulting in TATs exceeding two weeks [10]. In contrast, the Ion Torrent Genexus system used in this study achieved the following improvements: (1) simplified and automated workflow: streamlined the entire process from nucleic acid extraction to data interpretation; (2) reduced TAT: overall TAT was shortened to less than 27 h; (3) reduced hands-on time: manual work was limited to approximately 40 min; (4) cost efficiency: external testing and sample transport were eliminated; (5) feasibility in community hospitals: comprehensive genomic analysis was achieved for patients with limited access to tertiary centers; and (6) lower personnel burden: the system was operated by general laboratory staff without the need for advanced NGS expertise.

These improvements directly contribute to the optimization of cfDNA and ctDNA–based liquid biopsy workflows. Recent studies have similarly discussed the analytical performance and practical benefits of the Genexus platform in clinical molecular diagnostics [17,18,19]. For example, the Ion Torrent Genexus system has been shown to provide rapid and reproducible sequencing results through a fully automated process, supporting the broader implementation of precision oncology even in non-academic environments [20]. In addition, parallel evidence from digital PCR (dPCR)–based studies supports the complementary value of ctDNA monitoring for disease tracking and relapse detection in gastrointestinal cancers [21]. Collectively, these findings highlight the growing clinical relevance and reliability of automated cfDNA-based NGS workflows.

cfDNA extraction and library preparation in the present case were successfully performed using the Genexus Purification System and Oncomine Precision Assay GX5, which together enabled a true end-to-end automated workflow from sample to report. This approach removed multiple manual steps and substantially reduced operator-dependent variability. Previous reports have confirmed that the Genexus platform can deliver high-quality sequencing data comparable to those of conventional workflows while markedly reducing both TAT and hands-on time [10,19,20]. For instance, Sheffield et al. demonstrated that point-of-care implementation of the Genexus system at a community oncology center shortened the median TAT for molecular testing to within five business days [10]. Similarly, Krishnamurthy et al. validated the Oncomine Myeloid Assay GX v2 on the Genexus platform, confirming its analytical accuracy and robustness for detecting single-nucleotide variants, indels, and fusions in clinical diagnostics [19]. Moreover, Werner et al. reported the successful integration of a Genexus-based NGS workflow into an ISO 15189-accredited molecular diagnostics laboratory, further supporting its reproducibility and clinical reliability [20].

In the present case, cfDNA extraction and library preparation were successfully performed using the Genexus Purification System and OPA GX5 assay. According to a recent AACR 2024 evaluation, PAXgene^®^ tubes demonstrated minimal genomic DNA contamination and superior RNA performance compared with K_2_EDTA or Streck tubes, supporting their suitability for cfDNA-based workflows [16]. Furthermore, the use of PAXgene^®^ Blood ccfDNA tubes has been validated in colorectal cancer for highly sensitive detection of postoperative recurrence [11], reinforcing their reliability for cfDNA-based clinical workflows. This supports the technical robustness of the present workflow, even in a community hospital setting.

In addition to demonstrating the feasibility of an automated cfDNA-based NGS workflow, several technical considerations relevant to liquid biopsy should be addressed. First, user-dependent setup errors are a common limitation in semi-automated workflows; however, with the Genexus system, reagent handling, incubation timing, and thermal controls were standardized, minimizing operator-dependent variability. Second, hardware layout and consumable configuration—often sources of workflow optimization challenges—were predefined in this system, eliminating the need for extensive bench optimization. Third, the closed-system architecture substantially reduced opportunities for cross-contamination or PCR bias. Finally, because all critical steps are fully automated, inter-run variability was minimal, in contrast to multi-operator workflows that can introduce procedural inconsistency. Together, these features helped mitigate several well-recognized challenges in liquid biopsy-based NGS workflows.

A previous consensus statement by the International Association for the Study of Lung Cancer emphasized that plasma-based next-generation sequencing of ctDNA enables rapid molecular profiling during non-small cell lung cancer diagnosis and treatment, thereby supporting timely personalized therapeutic decisions [4]. In the same manner, in-house NGS testing at community hospitals may allow more patients to undergo regular monitoring with liquid biopsies, enabling earlier identification of molecular relapse and improved follow-up continuity. Furthermore, combining plasma-based and tissue-based molecular profiling may enhance diagnostic accuracy and strengthen the integration of genomic testing into real-world oncology practice [18].

Beyond the technical demonstration, the clinical relevance of cfDNA analysis has been increasingly recognized in colorectal and other solid tumors. Liquid biopsy-based detection of ctDNA enables sensitive monitoring for early recurrence and minimal residual disease MRD, complementing conventional radiologic assessment [22,23]. In colorectal cancer, ctDNA-guided strategies are being used to inform adjuvant therapy decisions and postoperative surveillance, with multiple prospective trials underway. These studies collectively demonstrate that ctDNA serves as a sensitive biomarker for early relapse detection, and that postoperative ctDNA positivity is strongly associated with poor recurrence-free survival, underscoring its prognostic and predictive value for MRD assessment [22,23]. Beyond colorectal cancer, cfDNA-based genomic profiling has demonstrated increasing clinical utility in pancreatic cancer, one of the most lethal malignancies. However, because the detected hotspot mutations are not associated with approved targeted therapies in pancreatic cancer, they are not used to guide systemic treatment. Clinical management follows standard therapeutic guidelines, while genomic findings provide supplementary molecular information and may potentially support molecular response monitoring during therapy. Recent reviews have emphasized that cfDNA plays a crucial role in the early diagnosis and personalized management of pancreatic cancer, serving as a promising biomarker for noninvasive disease detection and treatment monitoring [24]. Furthermore, genome-wide cfDNA sequencing approaches have been shown to enable real-time monitoring of therapeutic response, with molecular responders demonstrating significantly longer overall survival than nonresponders [25]. In addition, cfDNA-based liquid biopsy markers, including RAS mutation allele fraction, cfDNA concentration, and fragmentation index, have proven to be independent prognostic factors for overall and progression-free survival in metastatic pancreatic ductal adenocarcinoma, outperforming conventional serum markers, such as CA19-9, for disease monitoring [26]. The present case reinforces these findings by demonstrating that rapid cfDNA-based genomic profiling can be achieved within 27 h using a fully automated NGS workflow in a community hospital. This type of accelerated testing enables the timely identification of actionable genomic alterations and has the potential to facilitate early post-treatment molecular monitoring. Integration of rapid cfDNA-based sequencing workflows into daily oncology practice may therefore contribute to bridging the gap between advanced molecular diagnostics and community-based precision oncology.

Over the past decade, multiple community-based initiatives have explored the feasibility of implementing NGS outside academic centers to expand access to precision oncology. Early works, such as those reported by Akkari et al. in a mid-sized U.S. community hospital, underscored both the promise and the challenges of local NGS implementation [27]. The authors demonstrated that in-house testing could shorten diagnostic timelines but also highlighted key obstacles, including limited technical expertise, constrained bioinformatics infrastructure, and the need for institution-wide commitment to quality assurance. Together, these early efforts laid the groundwork for decentralizing molecular testing by demonstrating that community laboratories could successfully integrate genomic workflows once appropriate training and resources were in place.

Building on this foundation, Nicholas et al. reported the first real-world demonstration of a point-of-care liquid biopsy program within a Canadian community hospital using the Ion Torrent Genexus™ integrated sequencer and Oncomine Precision Assay GX5 [28]. They achieved a median TAT of only three business days from blood draw to report, proving that fully automated, in-house cfDNA sequencing can provide clinically actionable results with high sensitivity and reliability. This approach enabled oncologists to obtain genomic information rapidly to guide targeted therapy and resistance monitoring, thereby improving the timeliness of treatment decisions within a publicly funded healthcare system. In contrast, Fleming et al. reported that reliance on off-site molecular laboratories at a large regional cancer center prolonged biomarker reporting to a median of 36.5 days for non-squamous non–small cell lung carcinoma [29]. Only 20% of patients had complete molecular results available at first consultation, leading to delayed initiation of optimal systemic therapy and, in some cases, interim use of empiric chemotherapy or palliative radiotherapy. These findings emphasize how dependence on centralized testing infrastructure continues to limit equitable access to precision oncology for patients treated in community or regional settings. Taken together, these studies illustrate a clear transition from centralized, referral-based molecular diagnostics toward decentralized, community-based genomic testing. The present 27 h cfDNA-based workflow represents the next step in this evolution, demonstrating that fully automated NGS can be successfully implemented even in a community hospital environment. By eliminating external sample shipment and manual intervention, this approach not only minimizes human-dependent variability and cost but also enables near real-time delivery of clinically relevant results. Such innovation directly addresses the diagnostic inequality between tertiary and regional institutions and provides a practical model for integrating precision oncology into everyday clinical care.

Despite the encouraging results, this study has several limitations. First, it is based on a single case; therefore, the generalizability of the findings remains limited. Second, a formal cost evaluation was beyond the scope of this report, although future studies should assess the economic feasibility of system implementation. Finally, longitudinal evaluation across various cancer types is needed to confirm the robustness and scalability of the approach. Furthermore, standardization of sample handling, data processing, and interpretation across laboratories is critical to ensure reproducible outcomes. Integrating complementary high-sensitivity approaches, such as dPCR, may further enhance assay precision and confidence in variant detection. Indeed, individualized dPCR assays have already demonstrated clinical utility for frequent ctDNA monitoring in gastrointestinal cancers, enabling earlier relapse detection and optimized follow-up strategies compared with conventional imaging [21,30,31].

To facilitate future implementation, coordinated multi-institutional validation and health-economic evaluation will be essential for broader clinical adoption. Developing point-of-care and automated testing models adapted to regional healthcare policies could reduce dependence on centralized reference laboratories, thereby overcoming disparities in diagnostic accessibility [32]. Moreover, strengthening technical training for laboratory personnel could support consistent testing quality and sustainable implementation in community hospitals.

Looking forward, cfDNA-based NGS has the potential to transform precision oncology by enabling early detection, real-time monitoring of MRD, and rapid assessment of treatment response [31]. Such decentralized and automated molecular diagnostics may ultimately help bridge the gap between tertiary and community hospitals, advancing equitable access to personalized cancer care.

## 4. Conclusions

This case report demonstrated that rapid and comprehensive cfDNA-based genomic profiling can be completed within approximately 27 h by implementing a fully automated NGS workflow supported by an external reporting service in a community hospital. The workflow combined the Genexus™ Purification System and Genexus™ Integrated Sequencer for automated cfDNA extraction, library preparation, sequencing, and primary analysis. Subsequently, drug and clinical trial information were analyzed off-site, and a final report was generated through the Ion Torrent™ Cancer Genomic Analysis Report Service. This near-end-to-end process required less than 40 min of total hands-on time, eliminated the need for external sample shipment, and demonstrated the feasibility of generating clinically meaningful genomic results within a practical timeframe. By showing that high-quality molecular testing can be performed efficiently in a regional hospital environment, this study highlights a feasible pathway toward decentralizing precision oncology and bridging the diagnostic gap between tertiary and community healthcare facilities.

## Figures and Tables

**Table 1:** Detected genetic alterations identified by the automated NGS workflow.

	Sample Type	Gene Mutation (Variant)	COSMIC ID	Chromosome Position	Allele Frequency (%)	Copy Number Variation
Patient	Plasma (cfDNA, liquid biopsy)	*KRAS* p.(G12R), c.34G>C	COSM518	chr12:25398285	23.83	*PIK3CA* amplification (Copy number: 2.91)
		*TP53* p.(M246I), c.738G>C	COSM10757	chr17:7577543	0.27	
		*TP53* p.(M246I), c.738G>A	COSM44310	chr17:7577543	13.89	
		*TP53* p.(M246K), c.737T>A	COSM44103	chr17:7577544	31.32	
		*GNA11* p.(R183C), c.547C>T	COSM21651	chr19:3115012	0.09	
Healthy volunteer	Plasma (cfDNA, liquid biopsy)	None detected	–	–	–	None detected

Abbreviations: cfDNA, cell-free DNA.

**Table 2:** Summary of therapeutic associations and clinical trials identified through Oncomine™ Reporter analysis.

Gene Alteration	Therapeutic Agent/Combination	FDA	NCCN	EMA	ESMO	Clinical Trial Phase
PIK3CA amplification	Capivasertib + fulvestrant	●	×	×	×	×
	Atezolizumab + ipatasertib	×	×	×	×	(II)
	Temsirolimus	×	×	×	×	(II)
	Ipatasertib + atezolizumab	×	×	×	×	(I/II)
	Palbociclib + gedatolisib	×	×	×	×	(I)
KRAS G12R	Bevacizumab + CAPOX	×	×	×	●	×
	Bevacizumab + FOLFIRI	×	×	×	●	×
	Bevacizumab + FOLFOX	×	×	×	●	×
	Bevacizumab + FOLFOXIRI	×	×	×	●	×
	Atezolizumab + cobimetinib	×	×	×	×	(II)
	Regorafenib + trametinib	×	×	×	×	(II)
	Selumetinib + durvalumab	×	×	×	×	(II)
	Ulixertinib + antimalarial	×	×	×	×	(II)
	ZEN-3694 + talazoparib	×	×	×	×	(II)

Note: ○, approved or recommended for the same tumor type; ●, evidence in other tumor types; ×, no evidence available. Abbreviations: FDA, U.S. Food and Drug Administration; NCCN, National Comprehensive Cancer Network; EMA, European Medicines Agency; ESMO, European Society for Medical Oncology; CAPOX, capecitabine plus oxaliplatin; FOLFIRI, 5-fluorouracil, leucovorin, and irinotecan; FOLFOX, 5-fluorouracil, leucovorin, and oxaliplatin; FOLFOXIRI, 5-fluorouracil, leucovorin, oxaliplatin, and irinotecan.

## Data Availability

The data presented in this study are available from the corresponding author upon reasonable request.

## Abbreviations

The following abbreviations are used in this manuscript:

cfDNA	Cell-free DNA
CNV	Copy number variation
dPCR	Digital PCR
ctDNA	Circulating tumor DNA
GX5	Oncomine Precision Assay GX5
MRD	Minimal residual disease
NGS	Next-generation sequencing
TAT	Turnaround time
